# Solitary Rectal Ulcer Syndrome in Patients Presenting With Lower Gastrointestinal Bleeding: A Tertiary-Care Hospital Experience

**DOI:** 10.7759/cureus.35247

**Published:** 2023-02-21

**Authors:** Zubair Ejaz, Shafi U Khan, Rizwan U Rehman, Muhammad S Jibran

**Affiliations:** 1 Gastroenterology and Hepatology, Medical Teaching Institute Lady Reading Hospital, Peshawar, PAK; 2 Medicine, District Headquarter Hospital Kohat Develpment Authority, Kohat, PAK; 3 Gastroenterology, District Headquarter Hospital Kohat Development Authority, Kohat, PAK; 4 Cardiology, Medical Teaching Institue Lady Reading Hospital, Peshawar, PAK; 5 Cardiology, Mufti Mehmood Memorial Teaching Hospital Dera Ismail Khan, Dera Ismail Khan, PAK

**Keywords:** solitary rectal ulcer syndrome, rectal prolapse, ulcer, gastrointestinal diseases, gastrointestinal hemorrhage, lower gastrointestinal tract, constipation, anorectal disorders, rectal disease

## Abstract

Background: Solitary rectal ulcer syndrome (SRUS) is a benign rectal condition associated with defecation disorder that has multifactor pathologies and variable findings on presentation, endoscopy, and histopathology. A diagnostic dilemma with an overlap of differentials and step-wise management that starts with conservative therapies and goes up to repeated surgeries in case of failure of the conservative approach.

Objective: This study aims to observe clinical, endoscopic, and histological features of SRUS in patients presenting with lower gastrointestinal bleeding.

Material and methods: The study was conducted at the Department of Gastroenterology, Medical Teaching Institute, Lady Reading Hospital Peshawar from October 2018 to April 2020. After written informed consent, 257 patients (149 males and 108 females) from ages 15 to 70 who presented with lower GI bleeding were included via non-probability convenient sampling. Sociodemographic details were recorded in a pre-designed proforma. A colonoscopy was performed with the Colonoscope CF200 Z, Olympus Tokyo, Japan, and findings were noted. Suspected lesions were magnified, dyed with 0.2% indigo carmine, biopsied from the middle and edges of the ulcer, and sent for histopathology. All data were recorded and analyzed in SPSS-20. The mean with SD was calculated for quantitative variables, and frequency and percentages were calculated for qualitative variables. The chi-square test was used to check the significance, and a p-value of <0.05 was considered statistically significant.

Results: SRUS was found in 17 (6.6%) patients with lower GI bleeding, with a male predominance of 57% (n=11). Perirectal bleeding, constipation, mucous discharge, abdominal pain, and anemia were common clinical findings. Solitary lesions, ulceration, and anterior rectum location were the most common endoscopy findings. Obliterated lamina propria with collagen, ulceration, crypt distortion, and inflammatory infiltrates were common histopathological findings.

Conclusion: SRUS is a benign defecation disorder commonly presenting with lower GI bleeding, constipation, straining, and abdominal pain. It needs a stepwise approach with conservative management, medical management, biofeedback, and surgeries as a last resort.

## Introduction

Solitary rectal ulcer syndrome (SRUS) is a benign less common rectal condition, first time labeled in 1829 by Curveihier [1]. The term "solitary ulcer" was used for the first time in 1930 by Lloyd-Davis, and it was first widely reported by Madigan and Morson [2] in 1968 with 68 cases of SRUS. Since then it had many names like enterogenous cyst of the rectum, colitis cystica profunda, inverted hamartomatous polyp of the rectum, etc., in nomenclature [3]. Rutter and Riddell were the first to publish extensively regarding the pathogenesis of SRUS in 1975 [4].

Its average incidence reported in the literature is one in one hundred thousand people per year, which is underestimated as the SRUS is often missed [5,6]. It usually affects middle-aged adults and is less common in children. The commonest age group from 30 to 40 years is affected, with equal prevalence in men and women reported [7,8]. Common presentation findings are constipation, painful defecation, bleeding per rectum, straining, tenesmus, altered bowel habits, complete evacuation needing manual evacuation of stools, perianal pain, and rectal prolapse [3].

It is a spectrum of disorders with a misleading name, as not all lesions are ulcers, not all are confined to the rectum, and they vary in size, shape, and appearance from simple erythematous patches to polypoid or multiple ulcerative lesions. SRUS usually appears as typical shallow ulcerating lesions with hyperemic surroundings, mostly located on the anterior wall of the rectum about 3 to 10 cm from the anal verge [9]. Inflammatory bowel disease, infectious proctitis, ischemic colitis, latent mucosal prolapse of the rectum, rectal endometriosis, health anxiety disorders, obsessive-compulsive disorder, and malignant neoplasms, dyssynergic defecation disorders are some of the differentials of SRUS, which are differentiated by histopathological reports with the proliferation of smooth muscle fibers and obliteration of the lamina propria with collagen fibers as the most diagnostic markers of SRUS [8-10].

The treatment approach is stepwise and multidisciplinary, moving from conservative to interventional options like psychological, behavioral, physiological, pharmacological, and surgical [8]. Biofeedback therapies (BFT) targeting bowel training with techniques like time spent in the toilet, coordinating pelvic floor muscles and abdominal muscles for effective straining, supplementary use of Valsalva maneuvers, etc., have been reported to improve ulcer healing by up to 75% [11]. Some common surgeries used for surgical correction of SRUS are stapled transanal local excision (STARR) for paradoxical rectal spasm and complete internal prolapse, Altemeier perineal proctectomy for rectal excision, Delorme’s procedure for ulcer removal, and Rectopexy for rectal prolapse [12]. Perianal cerclage and sclerotherapy with submucosal and retrorectal injections of hypertonic saline, 25% glucose, or 5% phenol, and endoscopic application of human fibrin sealant have also been proven effective in some studies [13,14].

## Materials and methods

This monocentric study was performed at the Department of Gastroenterology and Hepatology, Medical Teaching Institute, Lady Reading Hospital, Peshawar, after receiving due approval from the ethical approval committee of the said institute. In this cross-sectional descriptive study, 257 patients between the ages of 15 and 70 were enrolled with lower gastrointestinal bleeding for at least one week via non-probability convenient sampling. The calculated sample size was 116 using the WHO sample size calculator, keeping the proportion of lower GI bleed in a solitary rectal ulcer at 82% [15], the general population proportion of 7.5% [16], a margin of error of 7%, and a confidence interval of 95%. However, to improve the study results, we enrolled 257 patients for colonoscopies. Patients with a diagnosed history of shock, visceral perforation, severe respiratory disease, and a recent history of myocardial infarction, unstable angina, or arrhythmias were excluded from the study. The duration of the study was from October 11, 2018, to April 11, 2020. Predesigned sociodemographic proformas were filled, history and physical examination was carried out, and a complete baseline workup like complete blood count, and hepatitis B, and C screening was done. Written informed consent was obtained. Patients were prepared for colonoscopy according to international guidelines. A 2-liter solution of electrolytes and polyethylene glycol was administered orally to all patients to prepare for a colonoscopy. A 20-mg intramuscular injection of buscopan was given after ruling out all possible contraindications to avoid spasms of the colon. All procedures for colonoscopy were performed with a video colonoscope of the model CF-200Z, Olympus Optical Company, Ltd., Tokyo, Japan. Both magnifying and conventional images were used. All lesions that were found at colonoscopy were magnified and dyed with indigo carmine 0.2% using chromoendoscopy. The sizes of the lesions were noted, and biopsies were taken both from the center and edges of the lesion and sent for histopathologies. Data regarding solitary rectal ulcer syndrome was noted, recorded, and confirmed with senior faculty members and supervisors. All the data collected were analyzed through the Statistical Package for Social Sciences (SPSS) version 24 (IBM Corp., Armonk, NY). Means and standard deviations were calculated for quantitative variables like age and duration of complaint. Frequency and percentages were calculated for qualitative variables like gender and solitary rectal ulcer syndrome. Stratification of age, gender, and duration was done against the solitary rectal ulcer syndrome, and the post-stratification chi-square test was applied to all variables with a p-value of less than 0.05 was considered statistically significant.

## Results

The study was performed on 257 patients (149 males and 108 females) who presented with lower gastrointestinal bleeding, of whom 17 patients (6.6%) were diagnosed with rectal ulcers on biopsies, with 11 males (64.7%) and 6 females (35.2%) with a gender ratio of 1.83 and male predominance. The mean age of the sample size was 39.8 ± 12.9 years. The mean duration of symptoms was 11.5 ± 4.3 weeks with a range from (1-23 weeks). The mean BMI was 26.3 (95% CI 19.6-32.7) in males and 24.7 in females (CI 95% 18.9-29.5), with an overall mean BMI of 25.5. The size of rectal ulcers ranged from 0.6 cm to 5.5 cm, with a mean value of 4.2 cm. The mean hemoglobin level was 8.8 mg/dl with a range of 4.3-15.6 mg/dl, with 5 (38%) mild to moderately anemic, 6 (46%) severely anemic, and 2 (11%) life-threateningly anemic. The distance of the lesion from the anal canal varied from 5 cm to 14 cm, with a mean distance of 8.8 cm. Table 1 shows all the detailed sociodemographic details of the study subjects.

**Table 1:** Socio demographic details of the study subjects (n=257)

Variable		Frequency	Percentage
Age	15-30 yrs	45	17.5%
	30-40 yrs	56	21.78%
	40-50 yrs	51	19.8%
	50-70 yrs	105	40.8%
	Total	257	100%
Gender	Male	149	57.97%
	Female	108	42.03%
	Total	257	100%
Duration of symptoms	1-4 week	60	23.3%
	4-8 weeks	81	31.5%
	8-12 weeks and above	116	45.13%
	Total	257	100%
Solitary rectal ulcer	Yes	17	6.8%
	No	240	93.3%
	Total	257	100%

Subjects between the ages of 30 and 40 were affected the most (p < 0.001). Males were predominantly affected (64.7%, n = 11), with a male-to-female ratio of 1.83 (p < 0.148). Most of the patients having SRUS were having symptoms duration of more than eight weeks (P < 0.040). Table 2 provides a detailed stratification of the variables of age, duration of symptoms, and gender of the study subjects.

**Table 2:** Stratification of solitary rectal ulcer syndrome with age, gender, and duration of symptoms

Variables		Solitary rectal ulcer syndrome					P-value
		Yes n=17		No n=240		Total	<0.001
		Frequency	Percentage	Frequency	Percentage		
Age	15–30 years	3	17.6%	42	17.5%	45	
	30–40 years	8	47%	48	20%	56	
	40–50 years	4	23.5%	47	19.58%	51	
	50–70 years	2	11.7%	103	42.9%	105	
	Total	17	100%	240	100%	257	
Gender	Male	11	64.7%	138	57.5%	149	0.148
	Female	6	35.2%	102	42.5%	108	
	Total	17	100%	240	100%	257	
Duration	1–4 weeks	2	11.7%	58	24.16%	60	0.040
	4–8 weeks	4	23.52%	77	32.08%	81	
	8–12 weeks or above	11	64.7%	105	43.75%	116	
	Total	17	100%	240	100%	257	

The most common presentations in history were bleeding per rectum 17 (100%), constipation 13 (76.4%), straining 8 (47%), mucous discharge 8 (47%), abdominal pain 11 (64%), tenesmus 2 (11%), and diarrhea 2 (11.7%). Commonly associated conditions were constipation 13 (76%), rectal prolapse 3 (17%), hemorrhoids 3 (17%), and digital rectal manipulation 3 (17%). Symptoms are mentioned in detail in Table 3. A P-value of less than 0.05 was considered significant.

**Table 3:** Symptoms of patients with solitary rectal ulcer syndrome (n=17)

Presentation	Number of patients (%)	P-value
Rectal bleeding	17 (100%)	<0.001
Straining	8 (47%)	<0.001
Abdominal pain	11 (64.7%)	<0.001
Pelvic fullness	4 (23.5%)	<0.01
Mucous discharge	8 (47%)	<0.001
Rectal prolapse	3 (17.6%)	<0.05
Incontinence	1 (5.08%)	<0.10
Urgency	3 (17.6%)	<0.05
Tenesmus	2 (11.7%)	<0.01
Incomplete/manual evacuation	3 (17%)	<0.05
Constipation	13 (76.4%)	<0.001
Diarrhea	2 (11.7%)	<0.1
Perianal pain	5 (29.4%)	<0.05
Altered bowel habits	5 (29.4%)	<0.05
Anemia	13 (76.4%)	<0.001
(1) Mild to moderate 8-12 g/dl	5 (38.4%)	
(2) Severe anemia 6 to 8 g/dl	6 (46.15%)	
(3) Life threatening anemia <6 g/dl	2 (11.7%)	
History of inflammatory bowel disease	1 (5.8%)	<0.1
History of bowel malignancies	1 (5.8%)	<0.1
Weight loss >5 kg in 6 months	2 (11.7%)	<0.1

On endoscopy, appearance-wise, 9 (52%) patients had solitary lesions, 4 (23%) had multiple ulcerations, 13 (76%) had ulcerative lesions, 3 (17%) had nodular/polypoid lesions, 3 (17%) had associated hemorrhoids, 4 (23.5%) had erythematous lesions, 13 (76.4%) had anterior lesions, 1 (5.08%) had a lateral lesion, 1 (5.08%) had a circumferential lesion, and 2 (11.7%) had posterior lesions, as shown in Table 4. P-values of less than 0.005 were considered significant.

**Table 4:** Endoscopy findings of patients with solitary rectal ulcer syndrome (n=17)

Endoscopy finding	Number of patients (%)	Significance (p-value)
Solitary lesion	9 (52.9%)	<0.001
Multiple lesions	4 (23.5%)	<0.01
Ulcerative	13 (76.4%)	<0.001
Nodular/polypoid	3 (17.6%)	<0.05
Hemorrhoids	3 (17.6%)	<0.05
Erythema only	4 (23.5%)	<0.01
Anterior lesions	13 (76.4%)	<0.001
Lateral lesions	1 (5.08%)	<0.10
Circumferential lesions	1 (5.08%)	<0.10
Posterior lesions	2 (11.7%)	<0.06

Histological features showed fibromuscular obliteration in 17 (100%), crypts distortion in 4 (23%), surface ulceration in 9 (52%), and inflammatory infiltrates in 10 (58.8%); 2 (11.7%) had crypts hyperplasia with diamond shapes, and 3 (17.6%) had mucosal gland distortion. Details are mentioned in Table 5. P-values of less than 0.05 were considered significant.

**Table 5:** Histopathology findings of patients with solitary rectal ulcer syndrome (n=17)

Histopathology findings	Number of patients (%)	P-value
Mucosal glands distortion	3 (17.6%)	<0.05
Fibromuscular obliteration	17 (100%)	<0.001
Superficial mucosal ulceration	9 (52.9%)	<0.001
Crypts distortion	4 (23.52%)	<0.05
Crypts hyperplasia and diamond shapes	2 (11.7%)	<0.10
Inflammation	10 (58.8%)	<0.001

All the patients were put on medical therapies and conservative management with diet, lifestyle, and biofeedback therapies and will be followed up for six months to see the response. Interventional approaches will be considered for patients with failure of conservative therapies or the presence of complications.

## Discussion

The diagnostic dilemma for solitary rectal ulcers is very common, and most ulcerated or hyperemic lesions are mistaken for inflammatory bowel diseases. Obliteration of fibromuscular tissue of the lamina propria is a very sensitive histological marker that differentiates between the two [17]. Overactivity of the anal sphincter has been reported to be an important pathophysiology factor by causing increased intrarectal pressure, increased transmural gradient, and increased voiding pressure to achieve satisfactory voiding that eventually leads to venous congestion and ulceration [18]. In a study conducted by Womack et al., the mean contraction of anal sphincters was 24 % higher in patients with SRUS compared to controls (p < 0.02), and mean intrarectal pressure was 74 mmHg in SRUS patients compared to controls of 44 mmHg (p < 0.02) [18]. Therefore, we think that patients with overactive anal sphincters who have SRU would benefit from biofeedback therapies for the correction of paradoxical contraction of external anal sphincters [11,18].

We also think that solitary rectal ulcer syndrome does not do justice to diverse presentations of the disease (polypoidal, nodular, multiple ulcers, hyperplastic polyps, erythematous lesions, telangiectasias, etc.), and a better nomenclature should be considered to bring all the possible variations under the umbrella of the diagnosis label [3,15].

A study by Zhu et al. [9] showed a predominance in the third and fourth decades of life, and our study also showed this age group to be affected more. However, in the case of women's predominance reported by Zhu et al., our study was quite the opposite and it showed more male predominance compared to females. Studies by Abusharifah et al. [19], Anjum et al. [20], and Dehghani et al. [21] also reported male predominance with 81%, 76.19%, and 74.5%, respectively. However, this fact needs to be followed up with larger sample sizes and preferably multicenter studies to find out if there is any gender predominance.

In all the literature available, rectal bleeding was the most common presentation, fibromuscular obliteration of the lamina propria was the most common histopathological finding, and the anterior wall of the rectum was the most common site. Our study was also in sync with the literature [8,15].

Our suggestions would be to first try conservative measures like reducing straining in defecation with good bowel habits, bowel training techniques with or without biofeedback, bulk laxatives (lactulose), enemas (mesalamine, sucralfate, steroids, etc.), oral 5-amino-salicylic acids, and endoscopic steroid injections. Above-mentioned measurements should be used, especially in people with no associated rectal prolapse, and local excisions should be considered only when symptoms are intractable. Surgeries such as rectopexy are recommended only when conservative management fails or there is associated rectal prolapse, both mucosal and full thickness [22-25]. Only large, solitary rectal ulcers should be considered for major resections with or without rectopexy. Ventral rectopexy is preferred for internal prolapse of the rectum, with good results and improvement of symptoms in up to 70% of cases and healing of ulcers in up to 90% of cases [26-28]. Older adults with multiple failed surgeries should be managed with fecal diversions. Tijandra et al. [3] have also recommended the above procedures. A recent consensus report for SRUS therapeutic management was also published, which endorses the stepwise management approach as mentioned above [29]. In some studies, novel therapies like argon plasma coagulation (APC) have been shown to be useful, especially in the treatment of bleeding ulcers, controlling them by 70% compared to medical therapies alone [30,31].

Some common symptoms like constipation, perianal pain, tenesmus, incomplete bowel emptying, straining, mucosal discharge, etc., are overlooked by physicians and gastroenterologists. We would suggest following up on these symptoms and getting a screening colonoscopy or sigmoidoscopy to get an early diagnosis of SRU if any, as our study subjects, like many other studies, have these common symptoms present in the histories of many patients. We would also suggest that all gastroenterologists should consider defecography, anorectal manometry, and endoscopic ultrasound more often in the above-mentioned scenarios to help find out early intussusception and rectal prolapse, as these tests help in the assessment of defecation disorders, incomplete evacuations, the relaxation status of puborectalis muscles, the thickness of internal sphincters, and occult prolapses [10,32]. So far, it is a big barrier due to the lack of availability and cost-effectiveness of the above-mentioned workup investigations.

## Conclusions

The incidence of solitary rectal ulcer syndrome was found to be low in our study, with a frequency of 6.6% (n=17). This benign defecation disorder commonly presented in our study with lower GI bleeding, constipation, straining, and abdominal pain. It needs a stepwise approach with conservative management, medical management, biofeedback, and surgeries as a last resort.
